# The role of dominant species in community organization and aboveground production in semiarid grasslands

**DOI:** 10.1002/ecy.70164

**Published:** 2025-08-11

**Authors:** Timothy J. Ohlert, Alesia Hallmark, Jennifer A. Rudgers, Debra P. C. Peters, Scott L. Collins

**Affiliations:** ^1^ Department of Biology University of New Mexico Albuquerque New Mexico USA; ^2^ Department of Biology Colorado State University Fort Collins Colorado USA; ^3^ Office of National Programs and the SCINet Big Data Program, United States Department of Agriculture Beltsville Maryland USA

**Keywords:** aboveground net primary production, biodiversity, biodiversity ecosystem function, *Bouteloua eriopoda*, *Bouteloua gracilis*, dominance, grassland, species richness

## Abstract

Dominant species play a key role in plant communities, influencing the abundance and richness of subordinate species through competitive and facilitative interactions. However, generalizations about the effects of dominant plant species in grasslands can be difficult due to the many differences among communities, such as abiotic conditions and regional species pools. To overcome this issue, we conducted a dominant species removal experiment in two semiarid grassland communities at the Sevilleta National Wildlife Refuge in central New Mexico. These communities had different dominant species but similar abiotic conditions and regional species pools. We studied the effects of removing dominant species on community composition, diversity, and aboveground net primary production (ANPP) over a 23‐year period. Our results showed that dominant grasses suppressed both richness and abundance of subordinate species. In the Chihuahuan Desert grassland, the loss of *Bouteloua eriopoda* was only partially compensated for by subordinate species, while in the Great Plains grassland, the loss of *Bouteloua gracilis* was fully compensated for after 16 years. Despite increased species richness, removing dominant species reduced ANPP and resulted in a negative relationship between species richness and ANPP in both grasslands. These results have important implications for ecosystem management and conservation, highlighting the potential impact of losing dominant species on subordinate species and community dynamics.

## INTRODUCTION

In most natural communities, a small number of species occur in high relative abundance among many less‐common and rare species (Alroy, 2015; Avolio et al., 2019; Gaston, 2011; Whittaker, 1965). As the most abundant species, dominants make a substantial contribution to ecosystem functioning, including nutrient cycling (Lohbeck et al., 2016; Suding et al., 2006), invasion resistance (Lyons & Schwartz, 2001; Smith et al., 2004; Souza et al., 2011), and aboveground net primary production (ANPP; Dee et al., 2019; Gaston, 2011; Genung et al., 2020). Theory suggests that the abundance of dominant species within a community drives ecosystem functioning more so than subordinate species, a phenomenon known as mass‐ratio effects (Grime, 1998; Smith et al., 2020). Therefore, highly dominant species should have the greatest impact on ANPP as their abundance drives total annual production (Avolio et al., 2019; van der Plas, 2019). In contrast, evidence is mixed regarding the degree to which loss of subordinate species affects ANPP, as some studies report ecological function loss with the decline of dominant species, while other studies find no change in ecological function. For example, production was maintained or increased in tallgrass prairie (Smith & Knapp, 2003), wet meadow (Lisner et al., 2023), and experimental grassland ecosystems when subordinate species were removed (Schmid et al., 2022). In other cases, a diverse set of subordinate species rapidly compensated for loss of dominant species (Allan et al., 2011; Wilcox et al., 2017; Zuppinger‐Dingley et al., 2014). In an alpine tundra, production was completely compensated by subordinate species 2 years after the removal of dominants (Suding et al., 2006) and subordinate species provided complete compensation within 1 year in a South African savanna grassland after the dominant grasses declined following a severe drought (Wilcox et al., 2020). Despite some examples of rapid recovery, loss of dominant species often results in long‐term, negative impacts on community production (Avolio et al., 2019; Munson & Lauenroth, 2009; Peters & Yao, 2012).

In addition to their effect on ANPP, dominant species often govern aspects of community structure such as species richness and evenness (Avolio et al., 2019; Ren et al., 2023; Souza et al., 2011). Dominant species can suppress subordinates through competition or promote them through facilitation (Ellison et al., 2005; Gaston, 2011). Results can be difficult to contextualize among studies, however, as differences in abiotic conditions and regional species pools confound comparisons. For example, removal of the dominant grass in a wet meadow increased species richness, attributed to reduced competition between dominant and subordinate species (Lepš, 2014). In environments with more evaporative demand, however, dominant species can facilitate growth of subdominant species by decreasing heat stress or altering soil moisture and nutrient levels (Bertness & Callaway, 1994; Lortie & Callaway, 2006; Wright et al., 2017). In addition, tall dominants can increase light limitation and thus reduce establishment and growth of short‐statured species (Eskelinen et al., 2022; Grman et al., 2021; Hautier et al., 2009) which may result in greater richness when dominant species are removed. In contrast, other studies have reported little or no effects of dominant species removal on diversity, even when ANPP declined (e.g., Li et al., 2015; Rixen & Mulder, 2009; Roth et al., 2008). Generalization across studies is difficult due to the myriad confounding variables that differ among sites, such as abiotic conditions and regional species pools. It is therefore important to contrast the effects of removal of different dominant species in communities with otherwise similar abiotic and biotic environments.

As anthropogenic stressors increasingly result in loss of dominant species (Castillioni et al., 2020; Jeger et al., 2014; Wilcox et al., 2020; Wilfahrt et al., 2020), greater importance is placed on understanding the potential loss of ecological function when dominants decline in a plant community. The degree to which subordinate species can compensate for the loss of dominants might be determined by the diversity and functional group of subordinate species. Given the well‐documented relationship between diversity and plant production (Hector et al., 1999; Tilman et al., 2014), an increase in species richness upon loss of dominant species could lead to greater compensation of production. Thus, the effects of dominant species on community composition, along with the secondary impacts of composition on ecosystem processes, including ANPP, may be linked to the number of species in the community following the removal of dominant species.

Varied conditions across study sites, including differences in competitive environments, resource availability, soil, and climate, make it challenging to generalize results across removal studies. Controlling for physical and biological variables, including climate and species pool, would improve understanding of how different dominant species impact ecosystem functioning. To this end, we used a long‐term (1995–2021) species removal experiment in two adjacent semiarid grasslands under similar soil and climate conditions to assess the capacity of plant communities to compensate for dominant species loss, as well as the importance of dominant species to annual production across a highly dynamic grassland ecotone (Collins et al., 2020). Our goal was to directly address the influence of dominant species on the diversity and production of dryland ecosystems in order to predict how anticipated changes in dominance will affect future production.

The co‐occurrence of these two grasslands with different dominant species under comparable environmental conditions creates the opportunity to determine the relative importance of species richness and dominance to aboveground production. We asked the following questions: (1) *How do different dominant plant species affect composition, diversity, and production?* Based on previous research, we expected that removal of the dominant species would alter community composition, increase species richness, and decrease production, but that the impacts of dominant removal on composition, diversity, and production would gradually decrease over time. (2) *Which species compensate in the absence of the dominant species?* Based on previous evidence of compensation by species within the same functional group as the dominant species (Buonopane et al., 2005; Chaves & Smith, 2021; Peters & Yao, 2012), we expected that subordinate grasses would increase in abundance upon removal of the dominant grasses. (3) *How does dominant species removal affect the species richness–ANPP relationship in these semiarid grasslands?* We expected that communities with greater species richness following the removal of dominant species would have more complete compensation for lost production.

## METHODS

### Study site

A long‐term dominant species removal experiment was conducted at the Sevilleta National Wildlife Refuge (SNWR) in central New Mexico, USA (34°20′ N, 106°43′ W). The SNWR is located at the transition zone between two ecosystems, Chihuahuan Desert grassland, which extends south into Mexico, and Great Plains grassland, which extends north into Colorado and east into Texas. Mean annual water year precipitation (MAP) is ~233 mm, with 51% falling from July to early September during the North American Monsoon (Brown & Collins, 2023; Pennington & Collins, 2007; Petrie et al., 2014). Mean monthly temperature ranges from 1.3°C in January to 24.5°C in July (Brown & Collins, 2023). At the study sites, ANPP is typically around 80 g/m^2^ in Great Plains grassland to 150 g/m^2^ in desert grassland (Appendix S1: Table S7). Soils are a sandy loam mixture including clay and calcium carbonate classified as Typic Haplargids at both sites (Kurc & Small, 2007; Ladwig et al., 2021; Peters & Yao, 2012).

The Chihuahuan Desert grassland is dominated by *Bouteloua eriopoda* (black grama), a stoloniferous, C4 perennial grass known to alter soil resources and increase colonization of subordinates (Schlesinger et al., 1999; Stewart et al., 2014; Zhang, 2021) and whose range extends southward through the arid regions of the Chihuahuan Desert in the United States and Mexico (Kröel‐Dulay et al., 2004). The Great Plains grassland is dominated by *Bouteloua gracilis* (blue grama), a long‐lived, caespitose, C4 perennial bunchgrass with small basal cover that typically forms rings in this system (Carlton et al., 2018; Coffin & Lauenroth, 1991; Peters, 2002; Ravi et al., 2008). A greater proportion of *B. gracilis* production is belowground compared to production of *B. eriopoda* (Gibbens & Lenz, 2001; Sun et al., 2024). Both grasslands feature several subdominant C4 grasses, including *Sporobolus* spp. and *Hilaria jamesii*. A mixture of shrubs and subshrubs can be found across the landscape, including *Yucca elata* and *Gutierrezia sarothrae*. Common forb species include the perennial *Machaeranthera pinnatifida* and the nonnative *Salsola tragus* (Mulhouse et al., 2017). Legumes are rare in these grasslands.

Previous studies have compared long‐term trends and vegetation dynamics of these grasslands in response to climate variability (Collins et al., 2020; Muldavin et al., 2008). Under increasing aridity, the Chihuahuan Desert grassland exhibits constrained aboveground production under wet conditions, while the Great Plains grassland is capable of large production increases in wet years (Rudgers et al., 2018). Both grasslands perform poorly under below‐average precipitation, and the Chihuahuan Desert grassland exhibits high sensitivity to short‐term drought (Knapp et al., 2015; Loydi & Collins, 2021). The dominant grasses account for over 80% of total ANPP in their respective ecosystems (Collins & Xia, 2015).

### Dominant species removal experiment

To determine the role of dominant species in the structure and production of these grasslands, we used a species removal experiment in Chihuahuan Desert and Great Plains grasslands (Peters, 2021). The distance between sites is 1 km across relatively flat topography. The experimental treatment involved removal of dominant species from their respective grasslands: *B. gracilis* in the Great Plains grassland and *B. eriopoda* in the Chihuahuan Desert grassland. In removal treatment plots, the dominant grasses were clipped or scraped along the soil surface annually with minimal soil disturbance (Peters & Yao, 2012). *B. gracilis* and *B. eriopoda* accounted for 25% and 22%, respectively, of absolute cover in control plots. In 2021, after 23 treatment years, *B. gracilis* and *B. eriopoda* accounted for only 5.5% and 0.7% absolute cover in removal plots in their respective grasslands before the annual removal treatment. Each site includes five removal treatment plots and five control plots within a 50 m by 50 m area; each plot is 3 m by 4 m. Percentage cover data for entire plots were collected at the species level. Using these observations of species percentage cover, we estimated aboveground biomass using allometric equations created between cover and biomass from plants harvested outside the permanent plots (Muldavin et al., 2008; Rudgers et al., 2019). Data were collected from 1995 to 2021 during peak production (September–October) following the summer monsoon season. We used the current year production as our measure of ANPP for herbaceous species, including grasses and forbs, which senesce each winter in these systems. ANPP for shrubs, cacti, yucca, and other woody species, including *G. sarothrae*, was calculated as current year standing biomass minus previous year standing biomass. In 1995 and 2001, plants were not identified to species, so species richness and community composition metrics were not attainable. Additionally, no data were collected in 2013, which prevented calculation of 2014 ANPP for shrubs and woody species.

### Statistical analysis

#### How do different dominant species affect composition, diversity, and production?

To test the effects of dominant species on community composition, we conducted a permutational multivariate ANOVA (PERMANOVA) using a Bray–Curtis dissimilarity matrix separately for each site with the adonis2 function in the vegan package (v2.6‐4; Oksanen et al., 2022) testing the effects of ecosystem type, removal treatment, and their interaction in the form community matrix ~ ecosystem × treatment. Observations for species with less than 1% cover were excluded. Each plot was used as a replicate, with plot and year included as random effects to control for repeated observations.

To assess the effects of dominant species removal on beta diversity, we first measured the average distance of replicates to the centroid of all replicates (i.e., group dispersion) for each treatment in each ecosystem in each year in Bray–Curtis space with the betadisper function in the vegan R package (v2.6‐4; Oksanen et al., 2022). We then tested the linear model: beta dispersion ~ ecosystem × treatment using the gls function and year in an autocorrelation structure using the corAR1 function in the nlme package (v3.1‐160; Pinheiro et al., 2022).

To test the effects of dominant species on species diversity, we quantified the species richness of subordinate species as the number of unique species present in each plot in each year, minus *B. gracilis* in the Great Plains control plots and *B. eriopoda* in the desert grassland control plots to make a more direct comparison since these species are removed from treatment plots. We then tested the linear model: subordinate species richness ~ year × treatment separately for each ecosystem using the lme function including plot as a random effect and year in an autocorrelation structure using the corAR1 function from the nlme package (v3.1‐160; Pinheiro et al., 2022). Pairwise comparisons of treatments in each year were obtained using the emmeans function in the emmeans package (v1.10.7; Lenth, 2025).

To test effects of dominant species on ANPP, we first summed the production of all species in each plot for every year of the study. Then, we tested the linear model: ANPP ~ year × treatment separately for each ecosystem using the lme function, including plot as a random effect and year in an autocorrelation structure using the corAR1 function from the nlme package (v3.1‐160; Pinheiro et al., 2022). Pairwise comparisons of treatments in each year were obtained using the emmeans function in the emmeans package (v1.10.7; Lenth, 2025).

#### Which species compensate in the absence of the dominant species?

To test which species increased in abundance following long‐term removal of the dominants, we performed indicator species analyses for each ecosystem using the multipatt function in the indicspecies R package (v1.7.14; De Cáceres & Legendre, 2009). Indicator species analyses (De Cáceres et al., 2010) were performed separately for each ecosystem in order to identify species abundances that were unique to the removal treatment in each ecosystem. Indicator values range from 0 to 1, where 1 represents a species that only occurs in a single group and is found at high abundance in all samples of that group. Based on the results of the indicator species analysis, which shows most compensation by subordinate grasses, we tested the compensation of the grass functional group with dominant removal. To do this, we summed the cover of grasses in each plot in each year and tested the linear model: grass cover ~ year × treatment separately for each ecosystem using the lme function, including plot as a random effect and year in an autocorrelation structure using the corAR1 function from the nlme package (v3.1‐160; Pinheiro et al., 2022). Pairwise comparisons of treatments in each year were obtained using the emmeans function in the emmeans package (v1.10.7; Lenth, 2025).

#### How does dominant species removal affect the species richness–ANPP relationship in these semiarid grasslands?

We summed the number of species and total biomass for each plot in each ecosystem in each year and tested the linear model: ANPP ~ species richness × treatment × ecosystem using the lme function including plot as a random effect and year in an autocorrelation structure using the corAR1 function from the nlme package (v3.1‐160; Pinheiro et al., 2022). We used the emtrends function in the emmeans R package (v1.10.7; Lenth, 2025) to compare the slope of species richness–ANPP among treatments and ecosystems.

We used R Statistical Software (v4.1.2; R Core Team, 2021) for all analyses. The tidyverse (v1.3.1; Wickham et al., 2019) and plyr (v1.8.6; Wickham, 2011) R packages were used for data manipulation and data visualization.

## RESULTS

### How do different dominant species affect composition, diversity, and production?

**FIGURE 1:**
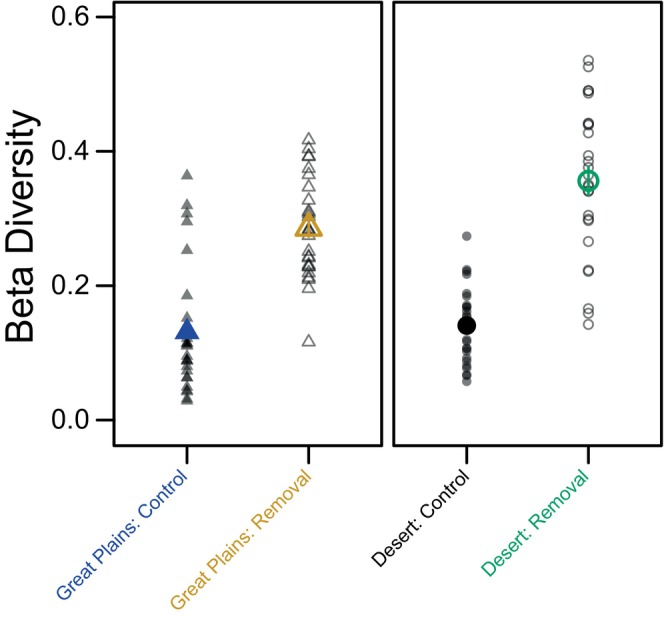
Beta diversity for grassland‐treatment combinations across all years of the experiment. For each year, average distance to centroid of plots in Bray–Curtis space was calculated for each grassland‐treatment combination shown as large points or triangles. Error bars denote SE. Beta diversity values for each year are shown in the background. Model results are presented in Appendix S1: Table S2.

Removal of dominant species altered both community composition and beta diversity of both grasslands. Community composition differed among sites (*p* < 0.001), treatments (*p* < 0.001), years (*p* < 0.001), and all two‐way interactions (Appendix S1: Table S1). In both grasslands, beta diversity was significantly greater in removal plots compared to controls (Great Plains estimate = 0.16, *p* < 0.001; Chihuahuan Desert estimate = 0.22, *p* < 0.01; Appendix S1: Table S2, Figure 1).

**FIGURE 2:**
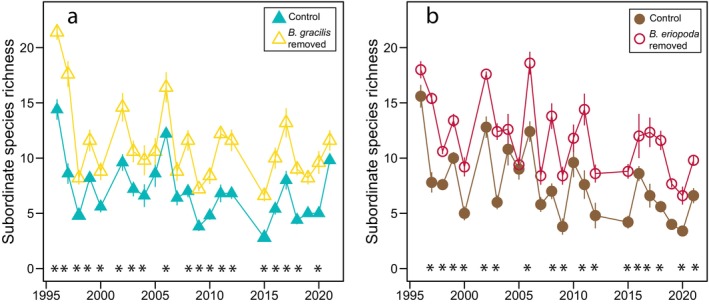
Comparison of subordinate species richness in control plots (filled points), and in plots where the dominant species was removed (open points) from 1995 to 2021 in (a) Great Plains grassland dominated by *Bouteloua gracilis* and (b) Chihuahuan Desert grassland dominated by *Bouteloua eriopoda* at the Sevilleta National Wildlife Refuge, New Mexico, USA. Points depict means and SE shown with lines around the means. Asterisks denote years in which the treatment and control plots were significantly different (*p* < 0.05) from each other based on pairwise comparisons on linear mixed‐effects models. Model results are presented in Appendix S1: Table S3.

**FIGURE 3:**
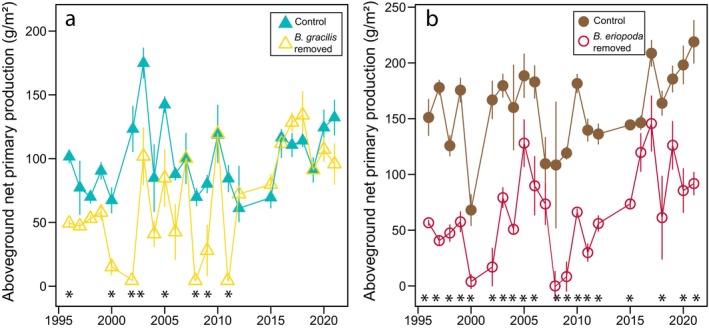
Comparison of aboveground net primary production (ANPP) in control plots (filled points), and in plots where the dominant species was removed (open points) from 1995 to 2021 in (a) Great Plains grassland dominated by *Bouteloua gracilis* and (b) Chihuahuan Desert grassland dominated by *Bouteloua eriopoda* at the Sevilleta National Wildlife Refuge, New Mexico, USA. Points depict means and SE shown with lines around the means. Asterisks denote years in which the treatment and control plots were significantly different (*p* < 0.05) from each other based on pairwise comparisons on linear mixed‐effects models. Model results are presented in Appendix S1: Table S4.

Subordinate species richness was higher in dominant species removal plots than in control plots in the Great Plains grassland over 19 years of the experiment and in the desert grassland over 17 years (Figure 2, Appendix S1: Table S3). The effect of dominant species removal on subordinate species richness remained consistent throughout the experiment, with years showing higher richness in removal plots compared to control plots being evenly distributed across the study period. In contrast, ANPP was greater in control plots than in dominant species removal plots during the first decade of the experiment in both the Great Plains grassland (five of the first 10 years) and desert grassland (seven of the first 10 years) (Figure 3, Appendix S1: Table S4). While these differences decreased in the Great Plains grassland, with only one significant difference in the last 10 years, they persisted in the desert grassland (seven of the last 10 years) (Figure 3, Appendix S1: Table S4).

### Which species compensate in the absence of the dominant species?

Removal of dominant species altered ANPP, with compensation primarily by subordinate grasses by the end of the experiment at both sites. Subordinate species had completely compensated for the loss of aboveground ANPP after the removal of *B. gracilis* in Great Plains grassland by 2011, while in the Chihuahuan Desert, subordinate species did not fully compensate for the loss of ANPP throughout the duration of the experiment (Figure 3). Furthermore, increased species richness was maintained in removal plots throughout the duration of the experiment (Figure 2).

In the Great Plains grassland, 12 plant species were useful indicators in differentiating removal treatment communities, while in the Chihuahuan Desert grassland, 15 species were useful indicators (Appendix S1: Table S8). Though both grasses and forbs served as indicator species, grasses generally had greater indicator values than forbs. Key species that differentiated the removal treatment in the Great Plains grassland included perennial grasses *H. jamesii*, *B. eriopoda*, and *Sporobolus cryptandrus*, as well as the forb *Sphaeralcea hastulata* (Appendix S1: Table S8). In the desert grassland, indicator species were generally less differentiating between the removal and control treatments (i.e., lower indicator values for desert grassland), but the most differentiating species were grasses *H. jamesii*, *Sporobolus contractus*, and *Bouteloua barbata* (Appendix S1: Table S8).

**FIGURE 4:**
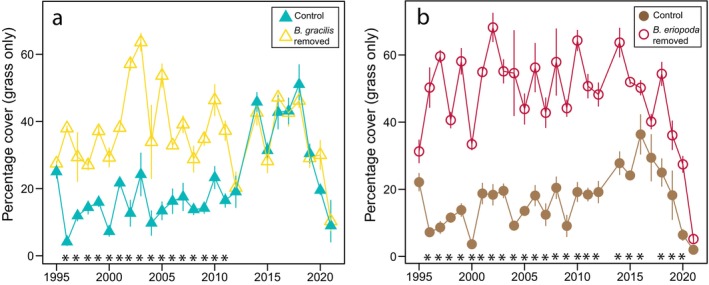
Percentage cover of grasses from 1995 to 2021. Points depict means and SE shown with lines around the means. Asterisks denote years in which the treatment and control plots were significantly different (*p* < 0.05) from each other based on pairwise comparisons on linear mixed‐effects models. Model results are presented in Appendix S1: Table S5.

Although grass cover initially decreased in both grasslands following the removal of dominant grasses, grass cover in the Great Plains grassland recovered midway through the experiment. From 2012 to the end of the study period, there were no differences in grass cover between treatment and control plots (Figure 4, Appendix S1: Table S5). In contrast, in the desert grassland, grass cover in the dominant species removal plots remained consistently lower than in control plots for eight of the last 10 years of the experiment (Figure 4, Appendix S1: Table S5).

### How does dominant species removal affect the species richness–ANPP relationship in these semiarid grasslands?

**FIGURE 5:**
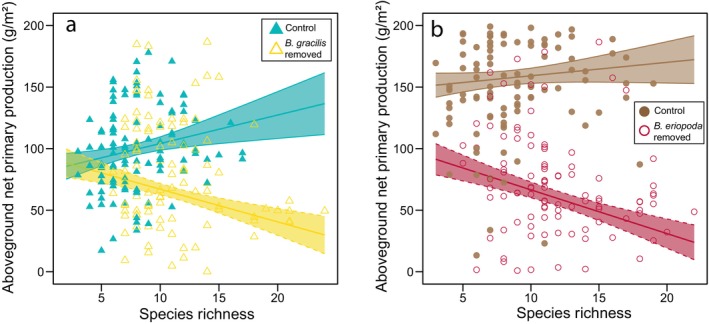
Relationship between species richness and aboveground net primary production (ANPP) in (a) Great Plains grassland and (b) Chihuahuan Desert grassland for control plots (solid points) and plots where dominant species were removed (open points). Model regressions and SEs are shown in blue and brown for control communities in Great Plains and Chihuahuan Desert, respectively, and yellow and red for treatment communities in Great Plains and Chihuahuan Desert, respectively. Model results are presented in Appendix S1: Tables S6 and S7.

In the absence of dominant species, we found a negative diversity‐productivity relationship in both grasslands. Under control conditions, there was no relationship between species richness and ANPP in the Great Plains grassland (*p* = 0.14, slope = 2.3, Figure 5, Appendix S1: Tables S6 and S7) nor Chihuahuan Desert grassland (*p* = 0.43, slope = 1.1, Figure 5, Appendix S1: Tables S6 and S7), although the slopes were positive at both sites. However, with the removal of dominant species, the relationship was significantly negative in both Great Plains grassland (*p* = 0.01, slope = −5.0) (Appendix S1: Tables S6 and S7, Figure 5) and Chihuahuan Desert grassland (*p* = 0.01, slope = −4.6, Figure 5, Appendix S1: Tables S6 and S7). Furthermore, the interaction effects of treatment and richness on ANPP show that the slope of the richness‐ANPP relationship decreased in the dominant removal treatment (*p* < 0.001; Appendix S1: Table S6) with a reduction of 4.1 g/m^2^ for every additional species in the removal treatment (*p* = 0.03, Appendix S1: Table S7).

## DISCUSSION

### Direct effects of dominant species on composition and diversity

Our 23‐year dominant species removal experiment in two semiarid grasslands under similar soil and climate conditions revealed the degree to which dominant grasses suppressed subordinate species richness and abundance. We also found that the removal of the dominant grasses in each community altered plant community composition and increased species richness and spatial heterogeneity (beta diversity), consistent with some previous studies (Avolio et al., 2019; Lepš, 2014; Souza et al., 2011). Our study also revealed that production by subordinate species gradually increased in these two grassland communities in the absence of the dominants. Increased beta diversity upon dominant removal suggests that the abundance of the dominant species greatly reduces spatial heterogeneity, likely through suppressed establishment and abundance of subordinate species. Although some prior studies showed that dominant removal can increase local species richness (Collins et al., 2002; Hernández et al., 2022; Suding et al., 2006), other experiments reported no effects of dominant removal on diversity (Raffaele & Ruggiero, 1995; Roth et al., 2008; Souza et al., 2011), indicating a lack of consistent response across systems. Disturbance in grasslands, such as drought, grazing, or fire, tends to promote greater species richness and more diverse communities when the disturbance disproportionately reduces the abundance of dominant species (Borer et al., 2014; Griffin‐Nolan et al., 2019; Grime, 2006; Koerner et al., 2018; Ladwig et al., 2014). Thus, the level of initial dominance and the degree to which disturbance reduces the abundance of dominants may explain disparate results among studies.

### Dominant species and ANPP

In both grasslands, removal of dominant species caused an initial reduction in total ANPP as expected, despite increased species richness. In Great Plains grassland, ANPP fully recovered after 16 years, whereas ANPP did not fully recover in Chihuahuan Desert grassland following removal of *B. eriopoda* after 23 years. Total production in Chihuahuan Desert grassland is higher than that of Great Plains grassland (Appendix S1: Table S5; Rudgers et al., 2018). Following dominant removal, plant communities in both sites converged onto a common set of subordinate species and ANPP of subordinate species increased to the same extent in both ecosystems. Thus, the lack of total recovery in Chihuahuan Desert grassland can be attributed to the higher contribution of *B. eriopoda* to total ANPP relative to that of *B. gracilis* in their respective undisturbed ecosystem. Although previous studies indicate that *B. gracilis* is a stronger competitor than *B. eriopoda* (Chung & Rudgers, 2016; Thomey et al., 2014), our results suggest that *B. eriopoda* is simply more productive under similar resource conditions. This result highlights important functional differences between these two closely related dominant grasses.

Our dominant removal experiment is one of the longest running such experiments in grassland ecosystems globally. Some previous removal experiments have shown partial or full compensation of production in response to dominant removal (Li et al., 2015; Pinder & John, 1975; Rixen & Mulder, 2009; Smith & Knapp, 2003; Souza et al., 2011). Of 16 prior studies that removed dominant plant species, 13 resulted in reduced ANPP over time. However, most of these studies were short term, with only three of the studies lasting 10 years. Incomplete compensation was evident in these longer term removal experiments. For example, removal of a dominant dwarf shrub in subalpine tundra led to decreased vascular plant cover over an 11‐year timeframe (Wardle et al., 2013) and removal of the dominant species from an alpine meadow decreased aboveground biomass for over 10 years (Akhmetzhanova, 2010). It is unclear if ANPP in these systems would eventually recover given more time. Clearly, more long‐term removal studies are needed to better understand and potentially generalize the consequences of loss of dominant species.

Earlier research found a negative effect of *B. eriopoda* removal on total vegetative cover in Chihuahuan Desert grassland, whereas total vegetative cover in the Great Plains grassland was restored after 10 years (Peters & Yao, 2012). However, initial recovery was mostly from forbs because of delays in the growth of subordinate grasses following the removal of the dominant grass in either grassland (Peters & Yao, 2012). After 23 years, however, indicator species analysis showed that compensation for the removal of dominant grasses in both grasslands was mostly attributable to other C4, perennial grasses more so than forbs. The slow increase by subordinate perennial grasses following dominant removal in both grasslands could be attributed to lag effects, plant–soil feedbacks, life history traits (e.g., clonality), nativeness, or a reliance on facilitation by the dominant species (Carlton et al., 2018; Chung et al., 2019; Chung & Rudgers, 2016; Ren et al., 2023). The fact that these differences in compensation across grasslands by subordinate grasses only appeared after 16 years further reinforces the need for multi‐decadal removal experiments to capture the full range of impacts due to the loss of dominant species.

### Species richness and mass‐ratio effects

The negative relationship between species richness and ANPP became more pronounced following the removal of dominant species, likely due to an increase in smaller plant species that contributed little to total ANPP. While subordinate perennial grasses compensated for much of the loss of dominant species, many of the species that increased in abundance after their removal were smaller forbs, such as *Pectis angustifolia*, *Nama hispidum*, and *S. hastulata*. In these grasslands, an increase in species richness driven by the addition of smaller forbs would have minimal impact on ANPP. Previous research demonstrates that rare species are less productive even in monoculture than abundant species (Parker et al., 2019) and the addition of rare species to communities has been shown to have a negative impact on ANPP across a global network of grasslands (Dee et al., 2023).

Although there was no significant relationship between species richness and ANPP under ambient conditions, the slopes tended to be positive, but the removal of dominant species resulted in significantly negative species richness–ANPP relationships in both grasslands. Recent studies have clarified the role of rare species in ecosystem function (Dee et al., 2019, 2023; Lisner et al., 2023; Schmid et al., 2022) and shown that mass‐ratio effects are more important than species loss in determining ecosystem responses to disturbance (Smith, 2011; Smith et al., 2020; Winfree et al., 2015). Importantly, the main effects of dominant species removal had a far greater effect on ANPP than species richness, which indicates strong mass‐ratio effects inherent in the dominant species of these semiarid grasslands. Since common and abundant species are particularly vulnerable to density‐dependent environmental stressors, projected increases in climate variability and frequency of drought in these grasslands could have a considerable impact on ANPP through their impacts on the dominant species, as opposed to their impacts on species richness or other measures of community structure (Rudgers et al., 2019).

Through this long‐term removal experiment, we demonstrated that mass‐ratio effects impose a stronger constraint on primary production than do species diversity in two adjacent semiarid grasslands dominated by different but closely related perennial grasses. Dominant species, while central to aboveground production, limit species richness even in systems where light does not limit coexistence (e.g., Eskelinen et al., 2022; Hautier et al., 2009). Compensation by subordinates was only partial at one site and occurred among functional groups, which unfolded over two decades of the experiment. Disturbances to these ecosystems, such as drought, fire, and grazing, are likely to disproportionately affect the abundances of the dominant grasses. For example, both dominants are highly susceptible to prolonged drought (Song et al., 2019) and *B. eriopoda* can take more than a decade to recover from wildfire (Brown & Collins, 2024). Such disturbances that diminish the production of the dominant species are among the greatest threats to ecosystem function in these grasslands. While management of these dominant species may occur at local scales, these species remain susceptible to regional‐scale drivers (e.g., Collins et al., 2020; Currier & Sala, 2022; Rudgers et al., 2018; Song et al., 2024) which are expected to be amplified with climate change (Ault et al., 2016; Cook et al., 2015). Thus, further efforts to buffer dominant species against disturbance provide the best opportunity to mitigate negative effects of anthropogenic global environmental change on ecosystems.

## AUTHOR CONTRIBUTIONS

Debra P. C. Peters designed the experiment. Timothy J. Ohlert analyzed the data and drafted the manuscript with Scott L. Collins. Alesia Hallmark contributed to data analysis and all authors contributed to writing and editing the manuscript.

## CONFLICT OF INTEREST STATEMENT

The authors declare no conflicts of interest.

## Supporting information


Appendix S1.

## Data Availability

Data (Ohlert & Hallmark, 2025) are available in EDI at https://doi.org/10.6073/pasta/e33cafd5b483b14e8b4e598089e6b60a and code (Ohlert, 2025) are available in Zenodo at https://doi.org/10.5281/zenodo.15596520.
