# Supplementary material for: The role of dominant species in community organization and aboveground production in semiarid grasslands
Source: Ecology. 2025 Aug 11;106(8):e70164. doi: 10.1002/ecy.70164 (PMC12340362; doi:10.1002/ecy.70164)
Supplement: Supplementary file 1 — Appendix S1. [file ECY-106-e70164-s001.pdf]

Appendix S1

Journal name: Ecology

Title: The role of dominant species in community organization and aboveground production in semiarid grasslands

Authors: Timothy J. Ohlert, Alesia Hallmark, Jennifer A. Rudgers, Debra P.C. Peters, Scott L. Collins

Supplemental tables

Table S1. Summary of PERMANOVA results testing effects of treatment (control or removal) and year of experiment on multivariate community composition for each site (Great Plains or Chihuahuan Desert grassland) separately. p values less than 0.05 are bolded.

Grassland	model term	df	sum of squares	R squared	F	p value
Great Plains	treatment	1	30.3	0.41	183.7	**0.001**
Great Plains	year	1	4.2	0.06	25.3	**0.001**
Great Plains	treatment *year	1	1.8	0.02	11.0	**0.001**
Chihuahuan Desert	treatment	1	25.4	0.34	140.5	**0.001**
Chihuahuan Desert	year	1	5.6	0.08	30.9	**0.001**
Chihuahuan Desert	treatment *year	1	0.5	0.01	2.9	**0.004**

Table S2. Comparison of beta diversity (plot dispersion) between treatments (control and removal) for each site separately. p values less than 0.05 are bolded.

Grassland	model term	value	standard error	df	t value	p value
Great Plains	Intercept	0.14	0.02	1	6.87	**0.0001**
Great Plains	Treatment	0.15	0.03	1	5.34	**0.0001**
Chihuahuan Desert	Intercept	0.15	0.04	1	4.05	**0.0002**
Chihuahuan Desert	Treatment	0.20	0.05	1	3.85	**0.0003**

Table S3. Comparison of species richness in each year at each site between treatments (control and removal). Negative estimates indicate where species richness was less in the removal treatment than control. p values less than 0.05 are bolded.

Grassland	year	estimate	SE	df	t.ratio	p.value
Great Plains	1996	-7	1.14	8	-6.12	**0.000282**
Great Plains	1997	-9	1.14	8	-7.87	**4.901295582 13556e-05**
Great Plains	1998	-3.4	1.14	8	-2.97	**0.017761**
Great Plains	1999	-3.4	1.14	8	-2.97	**0.017761**
Great Plains	2000	-3.2	1.14	8	-2.80	**0.02323**
Great Plains	2002	-5	1.14	8	-4.37	**0.002369**
Great Plains	2003	-3.4	1.14	8	-2.97	**0.017761**
Great Plains	2004	-3.2	1.14	8	-2.80	**0.02323**
Great Plains	2005	-2	1.14	8	-1.75	0.118333
Great Plains	2006	-4.2	1.14	8	-3.67	**0.006275**
Great Plains	2007	-2.4	1.14	8	-2.10	0.069009
Great Plains	2008	-4.6	1.14	8	-4.02	**0.003821**
Great Plains	2009	-3.4	1.14	8	-2.97	**0.017761**
Great Plains	2010	-3.6	1.14	8	-3.15	**0.013619**
Great Plains	2011	-5.4	1.14	8	-4.72	**0.001495**
Great Plains	2012	-4.8	1.14	8	-4.20	**0.003002**
Great Plains	2015	-3.8	1.14	8	-3.32	**0.010478**
Great Plains	2016	-4.6	1.14	8	-4.02	**0.003821**
Great Plains	2017	-5.2	1.14	8	-4.55	**0.001878**
Great Plains	2018	-4.6	1.14	8	-4.02	**0.003821**
Great Plains	2019	-3.2633	1.45	8	-2.24	0.055054
Great Plains	2020	-4.6	1.14	8	-4.02	**0.003821**
Great Plains	2021	-1.8	1.14	8	-1.57	0.154019
Chihuahuan Desert	1996	-2.4	1.28	8	-1.88	0.097268
Chihuahuan Desert	1997	-7.6	1.28	8	-5.95	**0.000344**

Grassland	year	estimate	SE	df	t.ratio	p.value
Chihuahuan Desert	1998	-3	1.28	8	-2.35	**0.046903**
Chihuahuan Desert	1999	-3.4	1.28	8	-2.66	**0.02881**
Chihuahuan Desert	2000	-4.2	1.28	8	-3.29	**0.011092**
Chihuahuan Desert	2002	-4.8	1.28	8	-3.76	**0.005583**
Chihuahuan Desert	2003	-6.4	1.28	8	-5.01	**0.001044**
Chihuahuan Desert	2004	-1.8	1.28	8	-1.41	0.196732
Chihuahuan Desert	2005	-0.4	1.28	8	-0.31	0.762337
Chihuahuan Desert	2006	-6.2	1.28	8	-4.85	**0.001271**
Chihuahuan Desert	2007	-2.6	1.28	8	-2.03	0.076377
Chihuahuan Desert	2008	-6.8	1.28	8	-5.32	**0.000711**
Chihuahuan Desert	2009	-4.6	1.28	8	-3.60	**0.006996**
Chihuahuan Desert	2010	-2.2	1.28	8	-1.72	0.123538
Chihuahuan Desert	2011	-6.8	1.28	8	-5.32	**0.000711**
Chihuahuan Desert	2012	-3.8	1.28	8	-2.97	**0.017794**
Chihuahuan Desert	2015	-4.6	1.28	8	-3.60	**0.006996**
Chihuahuan Desert	2016	-3.4	1.28	8	-2.66	**0.02881**
Chihuahuan Desert	2017	-6.23812	1.43	8	-4.35	**0.00245**
Chihuahuan Desert	2018	-6	1.28	8	-4.69	**0.001554**
Chihuahuan Desert	2019	-3.2302	1.43	8	-2.25	0.054413
Chihuahuan Desert	2020	-3.2	1.28	8	-2.50	**0.036745**
Chihuahuan Desert	2021	-3.2	1.28	8	-2.50	**0.036745**

Table S4. Comparison of aboveground net primary production (ANPP) in each year at each site between treatments (control and removal). Positive estimates indicate where ANPP was greater in control plots than removal plots. p values less than 0.05 are bolded.

Grassland	year	estimate	SE	df	t.ratio	p.value
Great Plains	1996	52.19918	19.82663	8	2.632781	**0.030048**
Great Plains	1997	30.00147	19.82663	8	1.51319	0.168688
Great Plains	1998	16.96947	19.82663	8	0.855893	0.416954
Great Plains	1999	32.57904	19.82663	8	1.643196	0.138966
Great Plains	2000	52.13388	19.82663	8	2.629487	**0.030202**
Great Plains	2002	129.7892	19.82663	8	6.546207	**0.000179**
Great Plains	2003	72.99877	19.82663	8	3.681854	**0.006203**
Great Plains	2004	43.7557	19.82663	8	2.206915	0.058361
Great Plains	2005	57.54453	19.82663	8	2.902385	**0.019819**
Great Plains	2006	45.22962	19.82663	8	2.281256	0.051969
Great Plains	2007	-0.162	19.82663	8	-0.00817	0.993681
Great Plains	2008	80.97273	19.82663	8	4.084038	**0.003514**
Great Plains	2009	52.15985	19.82663	8	2.630797	**0.030141**
Great Plains	2010	-0.83574	19.82663	8	-0.04215	0.96741
Great Plains	2011	161.9264	19.82663	8	8.167117	**3.76185223724309e-05**
Great Plains	2012	-11.1955	19.82663	8	-0.56467	0.587777
Great Plains	2015	-10.3297	19.82663	8	-0.521	0.616476
Great Plains	2016	4.551844	19.82663	8	0.229582	0.824177
Great Plains	2017	-14.801	22.70095	8	-0.652	0.532682
Great Plains	2018	-20.0654	19.82663	8	-1.01204	0.341144
Great Plains	2019	1.959573	25.84382	8	0.075824	0.941421
Great Plains	2020	17.13425	19.82663	8	0.864203	0.412637
Great Plains	2021	36.39654	19.82663	8	1.83574	0.103721
Chihuahuan Desert	1996	94.4159	25.15679	8	3.753098	**0.005599**
Chihuahuan Desert	1997	137.2838	25.15679	8	5.457127	0.000604
Chihuahuan Desert	1998	78.32572	25.15679	8	3.113502	0.014369

Grassland	year	estimate	SE	df	t.ratio	p.value
Chihuahuan Desert	1999	118.0158	25.15679	8	4.691209	0.001559
Chihuahuan Desert	2000	64.18874	25.15679	8	2.551547	**0.034092**
Chihuahuan Desert	2002	149.8496	25.15679	8	5.956624	**0.000339**
Chihuahuan Desert	2003	100.4526	25.15679	8	3.993063	**0.003988**
Chihuahuan Desert	2004	109.253	25.15679	8	4.342883	**0.002469**
Chihuahuan Desert	2005	60.42026	25.15679	8	2.401748	**0.043059**
Chihuahuan Desert	2006	93.143	25.15679	8	3.702499	**0.006021**
Chihuahuan Desert	2007	36.10776	25.15679	8	1.435309	0.189118
Chihuahuan Desert	2008	177.8585	25.15679	8	7.070001	**0.000105**
Chihuahuan Desert	2009	110.7942	25.15679	8	4.404146	**0.002274**
Chihuahuan Desert	2010	115.3396	25.15679	8	4.584829	**0.001791**
Chihuahuan Desert	2011	109.792	25.15679	8	4.364308	**0.002399**
Chihuahuan Desert	2012	80.26479	25.15679	8	3.190582	**0.012792**
Chihuahuan Desert	2015	70.89014	25.15679	8	2.817933	**0.022566**
Chihuahuan Desert	2016	26.69994	25.15679	8	1.061341	0.319522
Chihuahuan Desert	2017	64.86831	28.98098	8	2.238306	0.055572
Chihuahuan Desert	2018	102.6538	25.15679	8	4.080561	**0.003531**
Chihuahuan Desert	2019	55.93315	28.98098	8	1.929995	0.089721
Chihuahuan Desert	2020	112.6508	25.15679	8	4.477947	**0.002061**
Chihuahuan Desert	2021	127.0886	25.15679	8	5.051859	**0.000987**

Table S5. Comparison of grass cover in each year at each site between treatments (control and removal). Positive estimates indicate where grass cover was greater in control plots than removal plots. p values less than 0.05 are bolded.

Grassland	year	estimate	SE	df	t.ratio	p.value
Great Plains	1995	2.48	5.170446	8	0.479649	0.644315
Great Plains	1996	33.832	5.170446	8	6.543343	**0.00018**
Great Plains	1997	17.47	5.170446	8	3.378819	**0.009659**
Great Plains	1998	12.64	5.170446	8	2.444664	**0.040271**
Great Plains	1999	21.2	5.170446	8	4.100227	**0.003437**
Great Plains	2000	22.026	5.170446	8	4.259981	**0.002761**
Great Plains	2001	16.402	5.170446	8	3.17226	**0.01315**
Great Plains	2002	44.46	5.170446	8	8.598872	**2.58778912517681e-05**
Great Plains	2003	39.394	5.170446	8	7.619072	**6.19337811640982e-05**
Great Plains	2004	24.16	5.170446	8	4.672711	**0.001597**
Great Plains	2005	40.258	5.170446	8	7.786176	**5.30432047599836e-05**
Great Plains	2006	16.638	5.170446	8	3.217904	**0.012278**
Great Plains	2007	21.62	5.170446	8	4.181458	**0.003073**
Great Plains	2008	15.04	5.170446	8	2.90884	**0.019624**
Great Plains	2009	20.658	5.170446	8	3.9954	**0.003975**
Great Plains	2010	23.078	5.170446	8	4.463445	**0.002101**
Great Plains	2011	20.778	5.170446	8	4.018609	**0.003849**
Great Plains	2012	1.22	5.170446	8	0.235956	0.819395
Great Plains	2014	-3.14	5.170446	8	-0.6073	0.560491
Great Plains	2015	-3.16	5.170446	8	-0.61117	0.558052
Great Plains	2016	4.422	5.170446	8	0.855245	0.417292
Great Plains	2017	0.282081	5.876071	8	0.048005	0.962889
Great Plains	2018	-4.82	5.170446	8	-0.93222	0.378497
Great Plains	2019	1.399147	6.653522	8	0.210287	0.838701
Great Plains	2020	10.514	5.170446	8	2.03348	0.076443

Grassland	year	estimate	SE	df	t.ratio	p.value
Great Plains	2021	1.378	5.170446	8	0.266515	0.796584
Chihuahuan Desert	1995	9.15	5.587226	8	1.637664	0.140127
Chihuahuan Desert	1996	43.074	5.587226	8	7.709371	**5.69401569685002e-05**
Chihuahuan Desert	1997	50.93	5.587226	8	9.115436	**1.68721456754303e-05**
Chihuahuan Desert	1998	29.04	5.587226	8	5.19757	**0.000825**
Chihuahuan Desert	1999	44.28	5.587226	8	7.925221	**4.6719477748732e-05**
Chihuahuan Desert	2000	29.776	5.587226	8	5.329299	**0.000703**
Chihuahuan Desert	2001	36.186	5.587226	8	6.476559	**0.000193**
Chihuahuan Desert	2002	49.82	5.587226	8	8.916768	**1.98401797522572e-05**
Chihuahuan Desert	2003	35.592	5.587226	8	6.370245	**0.000216**
Chihuahuan Desert	2004	45.418	5.587226	8	8.1289	**3.89150602791953e-05**
Chihuahuan Desert	2005	30.362	5.587226	8	5.434181	**0.00062**
Chihuahuan Desert	2006	38.102	5.587226	8	6.819484	**0.000135**
Chihuahuan Desert	2007	30.358	5.587226	8	5.433465	**0.000621**
Chihuahuan Desert	2008	37.436	5.587226	8	6.700284	**0.000153**
Chihuahuan Desert	2009	35.058	5.587226	8	6.27467	**0.000239**
Chihuahuan Desert	2010	45.122	5.587226	8	8.075922	**4.07952677881369e-05**
Chihuahuan Desert	2011	32.28	5.587226	8	5.777464	**0.000416**
Chihuahuan Desert	2012	29.06	5.587226	8	5.20115	**0.000821**
Chihuahuan Desert	2014	35.922	5.587226	8	6.429308	**0.000203**
Chihuahuan Desert	2015	27.82	5.587226	8	4.979215	**0.001081**
Chihuahuan Desert	2016	13.878	5.587226	8	2.48388	**0.037882**
Chihuahuan Desert	2017	10.85716	6.224675	8	1.744213	0.119278
Chihuahuan Desert	2018	29.378	5.587226	8	5.258065	**0.000766**
Chihuahuan Desert	2019	16.90723	6.224675	8	2.716162	**0.026405**
Chihuahuan Desert	2020	21.02	5.587226	8	3.762153	**0.005527**
Chihuahuan Desert	2021	3.196	5.587226	8	0.572019	0.583021

Table S6. Summary of ANOVA from linear mixed effects model testing the main effects of species richness, treatment, and site as well as all two- and three-way interactions on ANPP. Plot was used as a random effect with year in an autocorrelation structure. Results are visualized in Figure 5. p values less than 0.05 are bolded.

	numDF	denDF	F-value	p-value
Intercept	1	427	1354.2	< **0.001**
Richness	1	427	42.1	< **0.001**
Treatment	1	16	130.4	< **0.001**
Site	1	16	29.1	< **0.001**
Richness:Treatment	1	427	13.1	< **0.001**
Richness:Site	1	427	8.1	< **0.01**
Treatment:Site	1	16	24.7	< **0.001**
Richness:Treatment:Site	1	427	0.1	0.75

Table S7. Model summary from linear mixed effects model testing the main effects of species richness, treatment, and site as well as all two- and three-way interactions on ANPP. Year was used as a random effect. Results are visualized in Figure 5. p values less than 0.05 are bolded.

Variable	Estimate	Standard error	DF	t-value	*p*
Intercept	152.4	12.8	427	11.9	**<0.001**
Richness	0.6	1.4	427	0.47	0.64
Removal	-51.9	20.4	16	-2.5	**0.02**
Site-Plains	-69.5	19.2	16	-3.6	**<0.01**
Richness x Removal	-4.1	1.9	427	-2.2	**0.03**
Richness x Site-Plains	1.4	2.1	427	0.67	**0.50**
Removal x Site-Plains	64.8	28.9	16	2.2	**0.04**
Richness x Removal x Site-Plains	-0.9	2.8	427	-0.32	0.75

Table S8. Indicator species analyses for the Great Plains and Chihuahuan Desert grasslands. Columns with species names denote the species that differentiate treatments for each grassland. Indicator value columns denote the explanatory power of each species to differentiate control and removal groups. Only species identified as significant indicator species are shown here.

Great Plains - Control	Great Plains - Removal	Indicator value		Chihuahuan Desert - Control	Chihuahuan Desert - Removal	Indicator value
Bouteloua gracilis		0.97		*Bouteloua eriopoda*		0.98
Machaeranthera pinnatifida		0.46			*Hilaria jamesii*	0.66
	Hilaria jamesii	0.91			*Sporobolus contractus*	0.60
	Sphaeralcea hastulata	0.84			*Bouteloua barbata*	0.60
	Bouteloua eriopoda	0.81			*Pectis angustifolia*	0.58
	Sporobolus cryptandrus	0.77			*Tidestromia lanuginosa*	0.56
	Pectis angustifolia	0.63			*Sporobolus cryptandrus*	0.50
	Sporobolus flexuosus	0.58			*Hymenopappus filifolius*	0.45
	Kallstroemia parviflora	0.46			*Euphorbia serrula*	0.40
	Opuntia phaeacantha	0.44			*Munroa squarrosa*	0.36
	Bouteloua barbata	0.41			*Dasyochloa pulchella*	0.34
	Tidestromia lanuginosa	0.41			*Astragalus sp.*	0.33
	Sphaeralcea incana	0.38			*Muhlenbergia torreyi*	0.31
	Munroa squarrosa	0.20			*Sphaeralcea hastulata*	0.29
					Nama hispidum	0.24
					Portulaca halimoides	0.22
